# Influenza D Virus in Animal Species in Guangdong Province, Southern China

**DOI:** 10.3201/eid2308.170059

**Published:** 2017-08

**Authors:** Shao-Lun Zhai, He Zhang, Sheng-Nan Chen, Xia Zhou, Tao Lin, Runxia Liu, Dian-Hong Lv, Xiao-Hui Wen, Wen-Kang Wei, Dan Wang, Feng Li

**Affiliations:** South Dakota State University, Brookings, South Dakota, USA (S.-L. Zhai, S.-N. Chen, T. Lin, R. Liu, D. Wang, F. Li);; Guangdong Academy of Agricultural Sciences, Guangzhou, China (S.-L. Zhai, D.-H. Lv, X.-H. Wen, W.-K. Wei);; South China Agricultural University, Guangzhou (H. Zhang, X. Zhou)

**Keywords:** influenza, influenza D virus, hemagglutinin-esterase-fusion gene, D/OK lineage, Holstein dairy cattle, yellow cattle, Asian buffalo, American Landrace pigs, hybrid goats, China, viruses, zoonoses

## Abstract

Molecular tests revealed influenza D viruses of D/OK lineage widely circulating in farmed animal species in Guangdong Province, southern China. In particular, we found high levels of influenza D virus infection in goats and pigs. We also detected viral RNA in serum specimens and feces of animals with certain severe diseases.

Four types of influenza viruses (A–D) have been confirmed (https://www.cdc.gov/flu/about/viruses/types.htm). The recently discovered influenza D virus is thought to cause respiratory diseases primarily in cattle and to a lesser extent in pigs (*1**–**4*). Moreover, serologic evidence for influenza D virus infection in small ruminants and humans has been established (*5**,**6*). Since the initial influenza D virus isolation in the United States in 2011 (*1*), the virus has been reported in China, Mexico, France, Italy, and Japan (*7**–**11*). Genetic analysis of the hemagglutinin-esterase-fusion gene demonstrated that these viruses had 2 distinct lineages, represented by D/OK and D/660 (*12*). Recently, a novel influenza D virus that emerged in Japan has been proposed as the third lineage (*11*). D/OK lineage–related viruses were previously identified in native Luxi yellow cattle in Shandong Province, northern China (*7*). Despite good progress in identifying domestic cattle as the primary reservoir of influenza D virus, we know little about prevalence in other animals. We conducted a study to clarify the origin and transmission dynamics of influenza D virus in goats, buffalo, and pigs as well as farmed cattle.

## The Study

**Figure 1:**
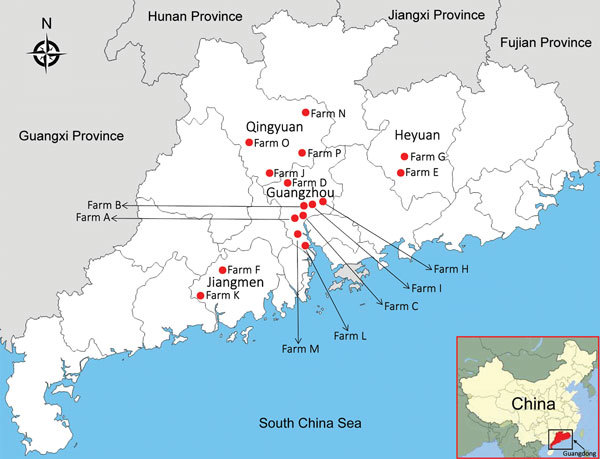
Farm locations for study of influenza D viruses in cattle, goats, buffalo, and pigs, Guangdong Province, China.

In 2016, we collected 607 clinical samples from 4 species of animals with different clinical diseases and 250 nasal swab samples from asymptomatic animals (Table) from 16 farms in 4 cities of Guangdong Province: Guangzhou, Qingyuan, Heyuan, and Jiangmen (Figure 1). In addition, we randomly chose 200 archived Holstein dairy cattle serum samples, 40 per year, from 2011–2015 to investigate possible early RNA distribution of influenza D virus in this region. We used the reverse transcription PCR method and subcloning protocol (Technical Appendix). We performed sequence alignment using ClustalW implemented in DNAStar software (DNAStar, Madison, WI, USA), and we conducted phylogenetic analyses based on our obtained sequences and reference truncated sequences (496-bp) of influenza D viruses from GenBank by using MEGA 5.1 software (http://www.megasoftware.net; Technical Appendix Table).

**Table:** Animal species, location, sample data, and detection rate of influenza D virus, Guangdong Province, China*

Animal species and farm	Farm type†	Farm location	No. animals	Age range of animals	Sample type	No. positive/no. samples	Detection rate, %
Holstein dairy cattle							
A	Not all-in-all-out	Guangzhou: Tianhe	2,000	3–5 y	Nasal swab	14/86‡	16.3
A	Not all-in-all-out	Guangzhou: Tianhe	2,000	3–5 y	Serum	10/94‡	10.6
B	Not all-in-all-out	Guangzhou: Luogang	800	3–6 y	Nasal swab	6/70‡	8.57
B	Not all-in-all-out	Guangzhou: Luogang	800	3–6 y	Serum	5/99‡	5.05
C	Not all-in-all-out	Guangzhou: Tianhe	175	2–5 y	Nasal swab	1/50§	2
American Landrace pig							
D	Not all-in-all-out	Guangzhou: Huadu	200	10–15 wks	Lung	4/10‡	40
E	All-in-all-out	Heyuan: Yuancheng	1,000	5–5 wks	Nasal swab	4/10‡	40
E	All-in-all-out	Heyuan: Yuancheng	1,000	3–5 wks	Lung	1/8‡	12.5
F	All-in-all-out	Jiangmen: Kaiping	800	8–20 wks	Nasal swab	3/9‡	30
F	All-in-all-out	Jiangmen: Kaiping	800	8–20 wks	Lung	8/27‡	29.6
G	All-in-all-out	Heyuan: Dongyuan	600	9–15 wks	Nasal swab	1/50§	2
Native hybrid white goat							
H	Not all-in-all-out	Guangzhou: Zengcheng	200	0.5–5 y	Serum	7/25‡	28
I	Not all-in-all-out	Guangzhou: Luogang	300	2–4 y	Serum	20/55¶	36.4
Native hybrid black goat							
J	Not all-in-all-out	Qingyuan: Jiangkou	150	1–3 y	Rectal swab	1/8#	12.5
K	Not all-in-all-out	Jiangmen: Enping	500	1–4 y	Nasal swab	0/50§	0
Asian buffalo							
L	Not all-in-all-out	Guangzhou: Nansha	150	3–5 y	Serum	2/26¶	7.7
M	Not all-in-all-out	Guangzhou: Panyu	180	3–6 y	Serum	1/25¶	4
N	Not all-in-all-out	Qingyuan: Yingde	400	1–4 y	Nasal swab	0/50§	0
Native yellow cattle							
O	Not all-in-all-out	Qingyuan: Qingxin	200	2–5 y	Nasal swab	4/55‡	7.3
P	Not all-in-all-out	Qingyuan: Fogang	230	1–3 y	Nasal swab	0/50§	0

*Feeding type of farms A–G was in captivity (poor biosecurity and high density). Feeding type of farms H–K and N–P was free grazing on the hills in the daytime and in captivity (poor biosecurity and high density) in the nighttime. Feeding type of farms L and M was free grazing in wetland in the daytime and in captivity (poor biosecurity and high density) in the nighttime. †All-in-all-out is a strategy for the control of infectious disease. The barn is emptied of all animals and the accommodation is cleaned and disinfected and then refilled, all on 1 day. ‡These animals had severe respiratory diseases with a 10%–30% mortality rate, mainly characterized by expiratory dyspnea and abdominal respiration. §These animals were asymptomatic. ¶These animals had severe reproductive disorders with a 60%–70% abortion rate. #These animals had severe diarrheal disease, characterized by watery diarrhea, limb weakness, and nearly dying.

After testing by reverse transcription PCR with further sequencing confirmation, we found influenza D virus–positive rates in 230 total nasal swab samples of 12.8% (20/156) for dairy cattle, 7.3% (4/55) for native yellow cattle, and 36.8% (7/19) for pigs. Rates in 324 total serum samples were 7.8% (15/193) for dairy cattle, 5.9% (3/51) for buffalo, and 33.8% (27/80) for goats. The influenza D virus–positive rate was also high (28.9%, 13/45) in swine lung samples. In contrast, we found no or low prevalence (<2%) in asymptomatic animals tested (Table). Moreover, all of the archived serum samples were found to be influenza D virus negative. Interestingly, 1 of 8 rectal swabs of goats with severe diarrhea tested positive (Table). Samples from animals with reproductive problems had a positive rate of 4.3% (5/116) (Table). 

**Figure 2:**
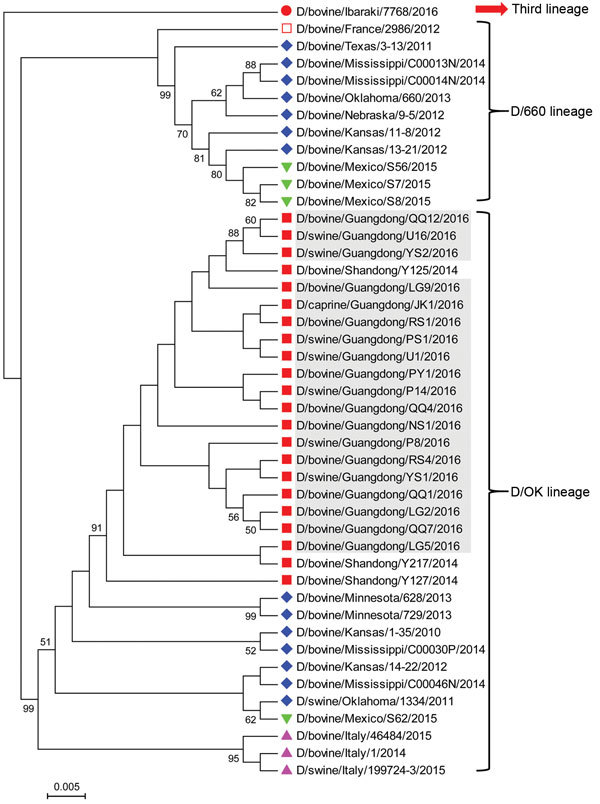
Phylogenetic analysis of viruses from study of influenza D viruses in cattle, goats, buffalo, and pigs in Guangdong Province and neighboring provinces, China, compared with reference viruses. Partial hemagglutinin-esterase-fusion gene sequences (496 bp) were aligned by using ClustalW implemented in DNAStar software (DNAStar, Madison, WI, USA), and the phylogenetic tree was obtained using neighbor-joining method within MEGA 5.1 software (http://www.megasoftware.net). Numbers at nodes are percentages of bootstrap values obtained by repeated analyses (1,000 times) to generate majority consensus tree. Only bootstrap scores of at least 50 were retained. Scale bar indicates 0.5% nucleotide sequence divergence. Gray shading indicates viruses from this study; reference viruses obtained from the United States are marked with ♦; from China, ■; from Italy, ▲; from Mexico, ▼; from France, □; and from Japan ●. Note that D/swine/Guangdong/YS1/2016 and D/swine/Guangdong/YS2/2016 are from the same farm; D/swine/Guangdong/P8/2016 and D/swine/Guangdong/P14/2016 are from the same farm; D/swine/Guangdong/U1/2016 and D/swine/Guangdong/U16/2016 are from the same farm; D/bovine/Guangdong/LG2/2016, D/bovine/Guangdong/LG5/2016 and D/bovine/Guangdong/LG9/2016 are from the same farm; D/bovine/Guangdong/QQ1/2016, D/bovine/Guangdong/QQ4/2016, D/bovine/Guangdong/QQ7/2016 and D/bovine/Guangdong/QQ12/2016 are from the same farm; D/bovine/Guangdong/RS1/2016 and D/bovine/Guangdong/RS4/2016 are from the same farm.

Sequence alignment analysis showed that the nucleotide sequences of influenza D viruses found in this study shared high similarity (99%–100%) with previously described sequences from China (*7*) and low similarity (93.8%–98.8%) with sequences originating from the United States, France, Italy, Mexico, and Japan (*1**,**8**–**12*). Similarly, phylogenetic analysis revealed that all influenza D virus sequences in this study clustered together with previous sequences from China and belonged to the D/OK lineage (Figure 2).

## Conclusions

When first discovered, influenza D virus was reported in diseased pigs in the United States (*1*). Later, it was identified in cattle and swine herds in several other countries, with or without clinical manifestation (*7**–**11*). Moreover, antibodies to influenza D virus were detected in goats, sheep, and humans (*5**–**6*). Under experimental conditions, influenza D virus replicated and transmitted among ferrets and guinea pigs (*13*). We confirmed that influenza D virus is widely present in cattle species (dairy cattle, yellow cattle, and buffalo). We also found influenza D virus at a high prevalence (>30%) in pigs and goats (Table), which is in contrast to the low prevalence found in previous investigations (*1**,**5**,**10*). The high prevalence may be caused by poor biosecurity measures and high-density feeding mode practices in China’s animal industry as well as possible cross-species transmission (*13*). Taken together, our findings expand the host range of influenza D virus and further emphasize the health concern this virus poses to multiple animal species.

Previous studies have shown that influenza D viruses are mainly found in respiratory tract samples (*1**–**4**,**7**,**9**–**12*) and that they have played an etiologic role in bovine respiratory diseases (*2**–**4*). In this study, we found that influenza D virus RNA was present in cattle and goat serum samples; it was also present in goat rectal swabs, accompanied by peste des petits ruminants virus and caprine kobuvirus (data not shown). The distribution of influenza D virus in our study is not the same as that described under experimental conditions (*3*). 

Influenza viremia, an indicator of disease severity (*14*), has been detected in 20.9% of severe cases during the acute phase of infection or before host death. Our detection of influenza D virus genome in serum samples from severely diseased animals (Table) implies that the virus could enter transiently into the animal’s circulatory system through capillaries lining the respiratory tract, which further contributes to the possibility of detecting virus in other organs. Similar to previous studies (*2**,**4*), we also found that the reverse transcription PCR positive rate was significantly higher (4%–40%) in diseased animals than the rate (<2%) observed in asymptomatic animals (p<0.05), which suggests a potential correlation between the disease severity and presence of influenza D virus. For influenza D virus found in rectal swabs, it might be that animals have swallowed the virus. Another possibility is that, similar to influenza A and B viruses, influenza D virus can replicate within the intestinal tract (*15*). 

We detected influenza D virus in cattle with reproductive disorders. However, we could not determine whether influenza D virus is associated with reproductive problems. Future studies can be designed to investigate these scientific issues.

To date, 2 lineages of influenza D virus (D/OK and D/660) co-circulate in North America and Europe (*8**–**10**,**12*). However, only the D/OK lineage has been found in China, and a potential third lineage was found in Japan (*7**,**11*). Our study confirms and further extends the previous observation that D/OK lineage circulates in East Asia. The viral, host, and ecologic factors that shape the observed contrasting phylodynamics of influenza D viruses among different geographic regions warrant further investigation. 

In addition, we found different minor genetic variants circulating on the same farm (Figure 2), indicating the ongoing evolution of influenza D viruses in their hosts (*7**,**8**,**11*). In comparing our sequences to the reference sequences from different animal species, we found 4 frequent nucleotide mutations (at positions 136, 231, 263, and 486) (online Technical Appendix Figure 1), which caused 2 amino acid mutations at positions 77 and 88 (online Technical Appendix Figure 2). Interestingly, among 4 nucleotide mutations, 1 unique nucleotide (T at position 486) was originally from the D/660 lineage. Moreover, we found several consistent sequences co-circulating in multiple animal species (online Technical Appendix Figure 1). Our speculation is that homologous recombination among different influenza D viruses and potential cross-species transmission under field conditions are possible, but further study is needed.

In summary, our study investigating the infection status of influenza D virus in different farmed animal species in Guangdong Province provides novel insights into the epidemiology and evolution of this virus. In particular, we document the molecular evidence for influenza D virus infection in goats and buffalo. 

Technical Appendix RT-PCR method and subcloning protocol used in study of influenza D viruses in cattle, goats, buffalo, and pigs, Guangdong Province and neighboring provinces, China.
