# Supplementary material for: Influenza D Virus in Animal Species in Guangdong Province, Southern China
Source: Emerg Infect Dis. 2017 Aug;23(8):1392–6. doi: 10.3201/eid2308.170059 (PMC5547803; doi:10.3201/eid2308.170059)
Supplement: Technical Appendix — RT-PCR method and subcloning protocol used in study of influenza D viruses in cattle, goats, buffalo, and pigs, Guangdong Province and neighboring provinces, China. [file 17-0059-Techapp-s1.pdf]

Article DOI: https://doi.org/10.3201/eid2308.170059

Influenza D Virus in Animal Species in Guangdong Province, Southern China

Technical Appendix

RT-PCR method and subcloning protocol used in this study

A classic reverse transcription PCR (RT-PCR) method was developed using a pair of primers (HEF-F: 5′-AAC CRC ATC TTC TTG TTC TTC A-3′ and HEF-R: 5′-TGC TTC TTC WGT GGC ATT ATC T-3′) targeting at the partial hemagglutinin-esterase-fusion (HEF) gene (at positions 582–1077) to test the presence of IDVs and further define IDV genetic lineages in those samples. For RT-PCR, the primers were diluted to 10 μM with ddH_2O.

A 25 μL RT-PCR system was constructed using a one-step RT-PCR kit (Takara Bio Inc.), which contained 0.5 μL of each primer, 0.5 μL of enzyme mixture (including PrimeScript RTase, DNA polymerase, RNase inhibitor), 12.5 μL of 2 × buffer, 8.5 μL of ddH_2O, and 2.5 μL of viral RNA. RT-PCR was performed as follows: reverse transcription (RT) at 50°C for 30 min, 95°C for 5 min, followed by 35 PCR cycles (95°C for 30 sec, 55°C for 30 sec, 72°C for 30 sec), and a final extension at 72°C for 10 min. The positive PCR fragments (≈500 bp) were purified and cloned into the pGEM–T vector (TIANGEN, Inc., Beijing). Positive recombinant plasmids were purified according to the manufacturer's instructions (TIANGEN, Inc., Beijing) and sequenced using the Sanger sequencing method (Sangon Biotech Co., Ltd., Shanghai). RT-PCR detection data were analyzed by chi-square test (Ziyue software), and significance was set at $p < 0.05$.

Technical Appendix Table. Reference influenza D virus isolates with partial hemagglutinin-esterase-fusion sequences used in this study

Strain or isolate	Specimen origin of isolate	Country	Year	GenBank accession no.
D/bovine/Nebraska/9–5/2012	Bovine (nasal swab)	USA	2012	KM392471
D/bovine/Oklahoma/660/2013	Bovine (nasal swab)	USA	2013	KF425662
D/bovine/Mississippi/C00014N/2014	Bovine (nasal swab)	USA	2014	KT581417
D/bovine/Mississippi/C00013N/2014	Bovine (nasal swab)	USA	2014	KT581416
D/bovine/Kansas/11–8/2012	Bovine (nasal swab)	USA	2012	KM392506
D/bovine/Kansas/13–21/2012	Bovine (nasal swab)	USA	2012	KM392492
D/bovine/Texas/3–13/2011	Bovine (nasal swab)	USA	2011	KM392485
D/bovine/Mexico/S56/2015	Bovine (nasal swab)	Mexico	2015	KU171128
D/bovine/Mexico/S8/2015	Bovine (nasal swab)	Mexico	2015	KU171127
D/bovine/Mexico/S7/2015	Bovine (nasal swab)	Mexico	2015	KU171126
D/bovine/France/2986/2012	Bovine (nasal swab)	France	2012	LN559126
D/bovine/Kansas/1–35/2010	Bovine (nasal swab)	USA	2010	KM392478
D/bovine/Mexico/S62/2015	Bovine (nasal swab)	Mexico	2015	KU171129
D/swine/Oklahoma/1334/2011	Swine (nasal swab)	USA	2011	JQ922308
D/bovine/Mississippi/C00030P/2014	Bovine (nasopharyngeal swab)	USA	2014	KT581418
D/bovine/Mississippi/C00046N/2014	Bovine (nasal swab)	USA	2014	KT581412
D/bovine/Minnesota/729/2013	Bovine (nasal swab)	USA	2013	KF425669
D/bovine/Minnesota/628/2013	Bovine (nasal swab)	USA	2013	KF425655
D/bovine/Kansas/14–22/2012	Bovine (nasal swab)	USA	2012	KM392499
D/bovine/Shandong/Y125/2014	Bovine (nasal swab)*	China	2014	KM015494
D/bovine/Shandong/Y127/2014	Bovine (nasal swab)*	China	2014	KM015501
D/bovine/Italy/46484/2015	Bovine (nasal swab)	Italy	2015	KT592526
D/bovine/Italy/1/2014	Bovine (nasal swab)	Italy	2014	KT592522
D/bovine/Shandong/Y217/2014	Bovine (nasal swab)*	China	2014	KM015508
D/swine/Italy/199724–3/2015	Swine (nasal swab)	Italy	2015	KT592533
D/bovine/Ibaraki/7768/2016	Bovine (nasal swab)	Japan	2016	LC128433
D/swine/Guangdong/YS1/2016	Swine (lung)	China	2016	KY441104
D/swine/Guangdong/YS2/2016	Swine (lung)*	China	2016	KY441105
D/swine/Guangdong/P8/2016	Swine (lung)	China	2016	KY441106
D/swine/Guangdong/P14/2016	Swine (nasal swab)	China	2016	KY441107
D/swine/Guangdong/PS1/2016	Swine (nasal swab)*	China	2016	KY441108
D/swine/Guangdong/U1/2016	Swine (serum)	China	2016	KY441109
D/swine/Guangdong/U16/2016	Swine (nasal swab)*	China	2016	KY441110
D/bovine/Guangdong/LG2/2016	Bovine: dairy cow (serum)	China	2016	KY441111
D/bovine/Guangdong/LG5/2016	Bovine: dairy cow (nasal swab)	China	2016	KY441112

Strain or isolate	Specimen origin of isolate	Country	Year	GenBank accession no.
D/bovine/Guangdong/LG9/2016	Bovine: dairy cow (nasal swab)*	China	2016	KY441113
D/bovine/Guangdong/QQ1/2016	Bovine: dairy cow (nasal swab)	China	2016	KY441114
D/bovine/Guangdong/QQ4/2016	Bovine: dairy cow (serum)	China	2016	KY441115
D/bovine/Guangdong/QQ7/2016	Bovine: dairy cow (serum)	China	2016	KY441116
D/bovine/Guangdong/QQ12/2016	Bovine: dairy cow (nasal swab)*	China	2016	KY441117
D/bovine/Guangdong/NS1/2016	Bovine: buffalo (serum)	China	2016	KY441118
D/bovine/Guangdong/RS1/2016	Bovine: yellow cattle (nasal swab)	China	2016	KY441119
D/bovine/Guangdong/RS4/2016	Bovine: yellow cattle (nasal swab)*	China	2016	KY441120
D/bovine/Guangdong/PY1/2016	Bovine: buffalo (serum)	China	2016	KY441121
D/caprine/Guangdong/JK1/2016	Caprine: goat (rectal swab)	China	2016	KY441122

*Healthy.

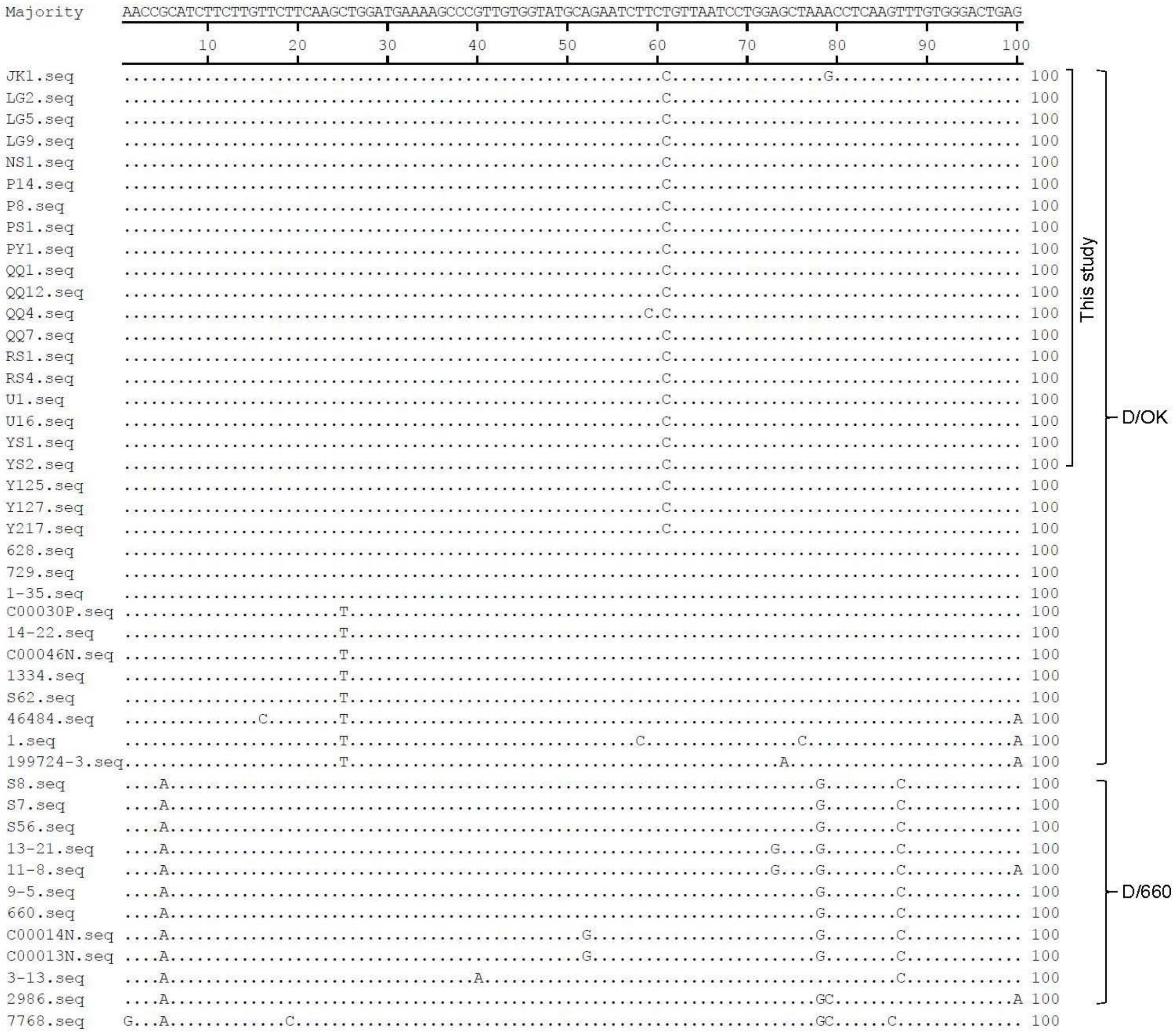

Majority
AACCGCATCTTCTTGTTCTTCAAGCTGGATGAAAAGCCCGTTGTGGTATGCAGAATCTTCTGTTAATCCTGGAGCTAAACCTCAAGTTTGTGGGACTGAG
10
20
30
40
50
60
70
80
90
100
JK1.seq
LG2.seq
LG5.seq
LG9.seq
NS1.seq
P14.seq
P8.seq
PS1.seq
PY1.seq
QQ1.seq
QQ12.seq
QQ4.seq
QQ7.seq
RS1.seq
RS4.seq
U1.seq
U16.seq
YS1.seq
YS2.seq
Y125.seq
Y127.seq
Y217.seq
628.seq
729.seq
1-35.seq
C00030P.seq
14-22.seq
C00046N.seq
1334.seq
S62.seq
46484.seq
1.seq
199724-3.seq
S8.seq
S7.seq
S56.seq
13-21.seq
11-8.seq
9-5.seq
660.seq
C00014N.seq
C00013N.seq
3-13.seq
2986.seq
7768.seq
This study
D/OK
D/660

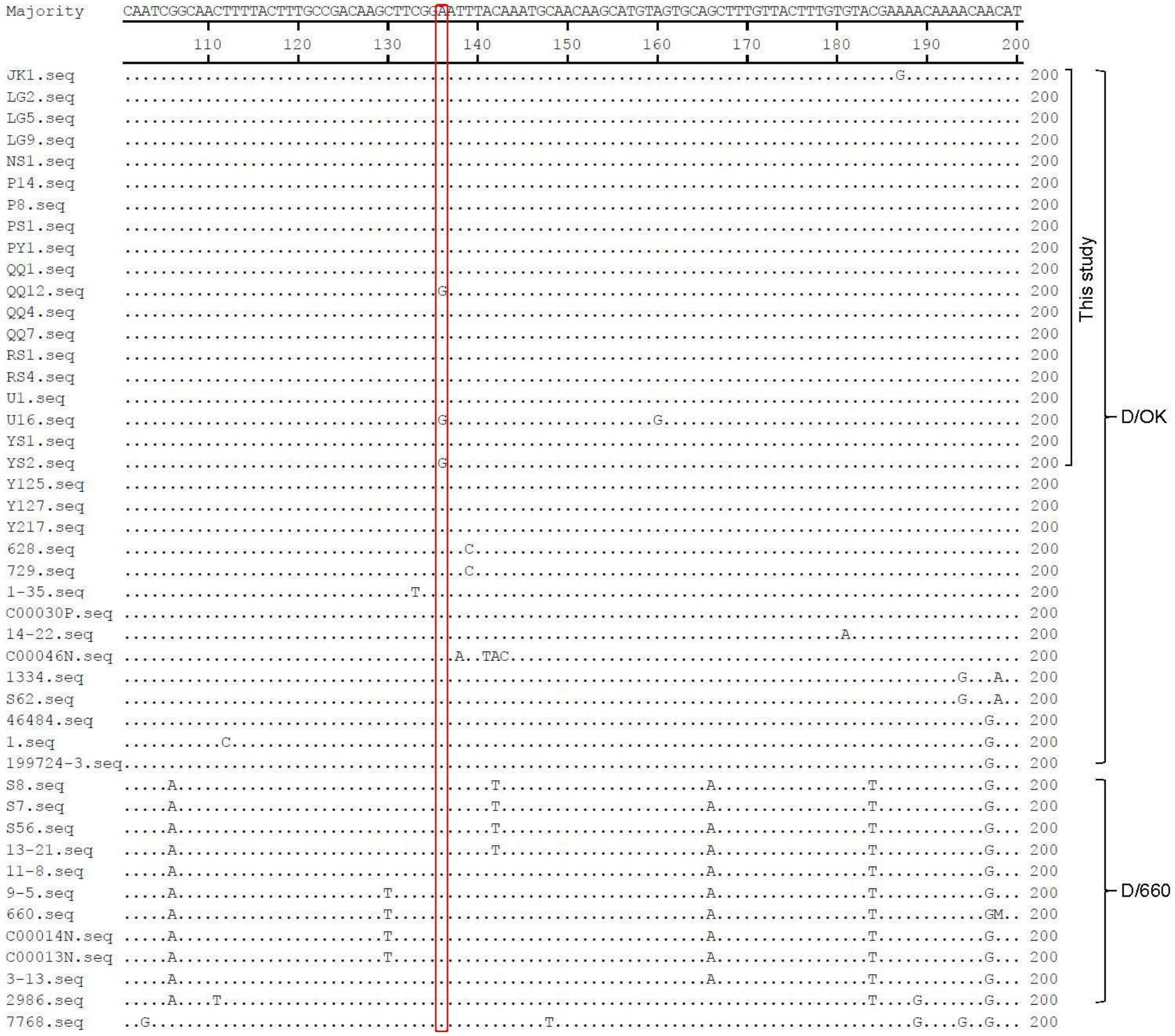
Majority
CAATCGGCAACTTTTACTTTGCCGACAAGCTTCGGAATTTACAAATGCAACAAGCATGTAGTGCAGCTTTGTTACTTTGTGTACGAAAACAAAACAACAT
This study
D/OK
D/660

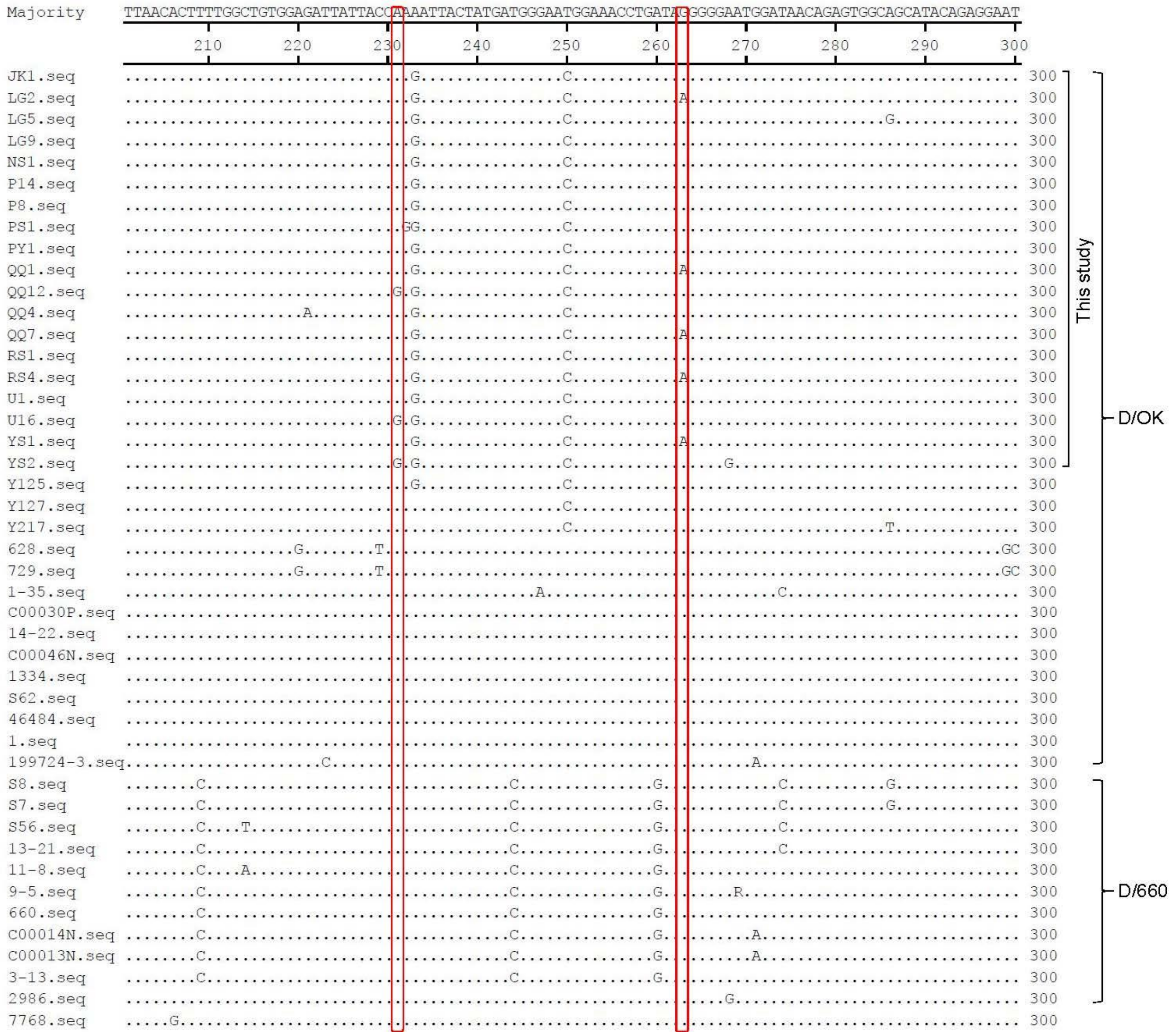
Majority
TTAACACTTTTGGCTGTGGAGATTATTACCAAAATTACTATGATGGGAATGGAAACCTGATAGGGGGAATGGATAACAGAGTGGCAGCATACAGAGGAAT
210 220 230 240 250 260 270 280 290 300
JK1.seq
LG2.seq
LG5.seq
LG9.seq
NS1.seq
P14.seq
P8.seq
PS1.seq
PY1.seq
QQ1.seq
QQ12.seq
QQ4.seq
QQ7.seq
RS1.seq
RS4.seq
U1.seq
U16.seq
YS1.seq
YS2.seq
Y125.seq
Y127.seq
Y217.seq
628.seq
729.seq
1-35.seq
C00030P.seq
14-22.seq
C00046N.seq
1334.seq
S62.seq
46484.seq
1.seq
199724-3.seq
S8.seq
S7.seq
S56.seq
13-21.seq
11-8.seq
9-5.seq
660.seq
C00014N.seq
C00013N.seq
3-13.seq
2986.seq
7768.seq
This study
D/OK
D/660

```
Majority     AGCAAACGCTGGAGTTAAAATTGAATGTCCTTCCAAAATCTTGAACCCTGGGACTTACAGCATTAGATCAACACCAAGATTCCTTCTAGTACCAAAAAGG
                      310       320       330       340       350       360       370       380       390       400
JK1.seq      .................................................................................................... 400
LG2.seq      .A.................................................................................................. 400
LG5.seq      .................................................................................................... 400
LG9.seq      .................................................................................................... 400
NS1.seq      .................................................................................................... 400
P14.seq      .................................................................................................... 400
P8.seq       .................................................................................................... 400
PS1.seq      .................................................................................................... 400
PY1.seq      .................................................................................................... 400
QQ1.seq      .................................................................................................... 400
QQ12.seq     .................................................................................................... 400  This study
QQ4.seq      .................................................................................................... 400
QQ7.seq      .................................................................................................... 400
RS1.seq      .................................................................................................... 400
RS4.seq      .................................................................................................... 400
U1.seq       .................................................................................................... 400
U16.seq      .................................................................................................... 400  D/OK
YS1.seq      .................................................................................................... 400
YS2.seq      .................................................................................................... 400
Y125.seq     .................................................................................................... 400
Y127.seq     .................................................................................................... 400
Y217.seq     .................................................................................................... 400
628.seq      ..............................................................................................G...... 400
729.seq      ..............................................................................................G...... 400
1-35.seq     .......A............................................................................................ 400
C00030P.seq  .......A............................................................................................ 400
14-22.seq    ....................................................................................C............... 400
C00046N.seq  .................................................................................................... 400
1334.seq     .................................................................A.................................. 400
S62.seq      .................................................................A.................................. 400
46484.seq    ........T........................................................................................... 400
1.seq        ........T........................................................................................... 400
199724-3.seq ........T....................................T...................................................... 400
S8.seq       ....GG.T.........G...............A.........................................C.A.........G............ 400
S7.seq       ....GG.T.........G...............A.........................................C.A.........G............ 400
S56.seq      ....GG.T.........................A....................C....................C.A.........G............ 400
13-21.seq    ....GG.T.........G...............A........................C..................A.........G............ 400
11-8.seq     ....GG.T.........................A...........................................A.........G............ 400
9-5.seq      ....GG.T.........................A...............................R...........A.........G............ 400  D/660
660.seq      ....GG.T.........................A...........................................A.........G............ 400
C00014N.seq  ....GG.T.........................A...........................................A.........G............ 400
C00013N.seq  ....GG.T.........................A...........................................A.........G............ 400
3-13.seq     ....GG.T..R......................A...........................................A......G..G..G......... 400
2986.seq     ....GGT.......C........................T........A................................................... 400
7768.seq     .....G.....A................................G..............................G.A.........G............ 400
```

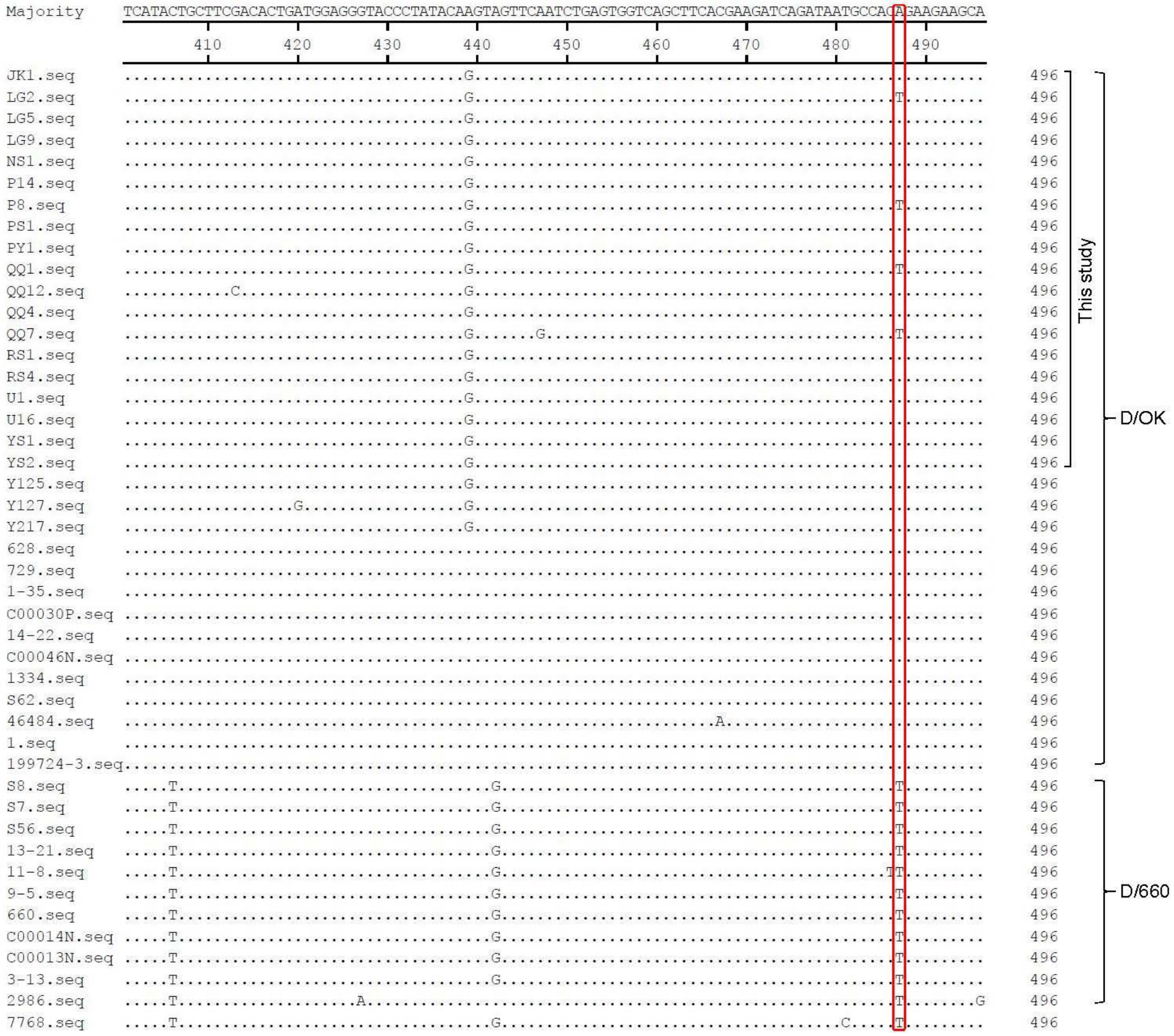

Technical Appendix Figure 1. Multiple sequence alignment results of the current IDV nucleotide sequences (496 bp) and corresponding published reference sequences.

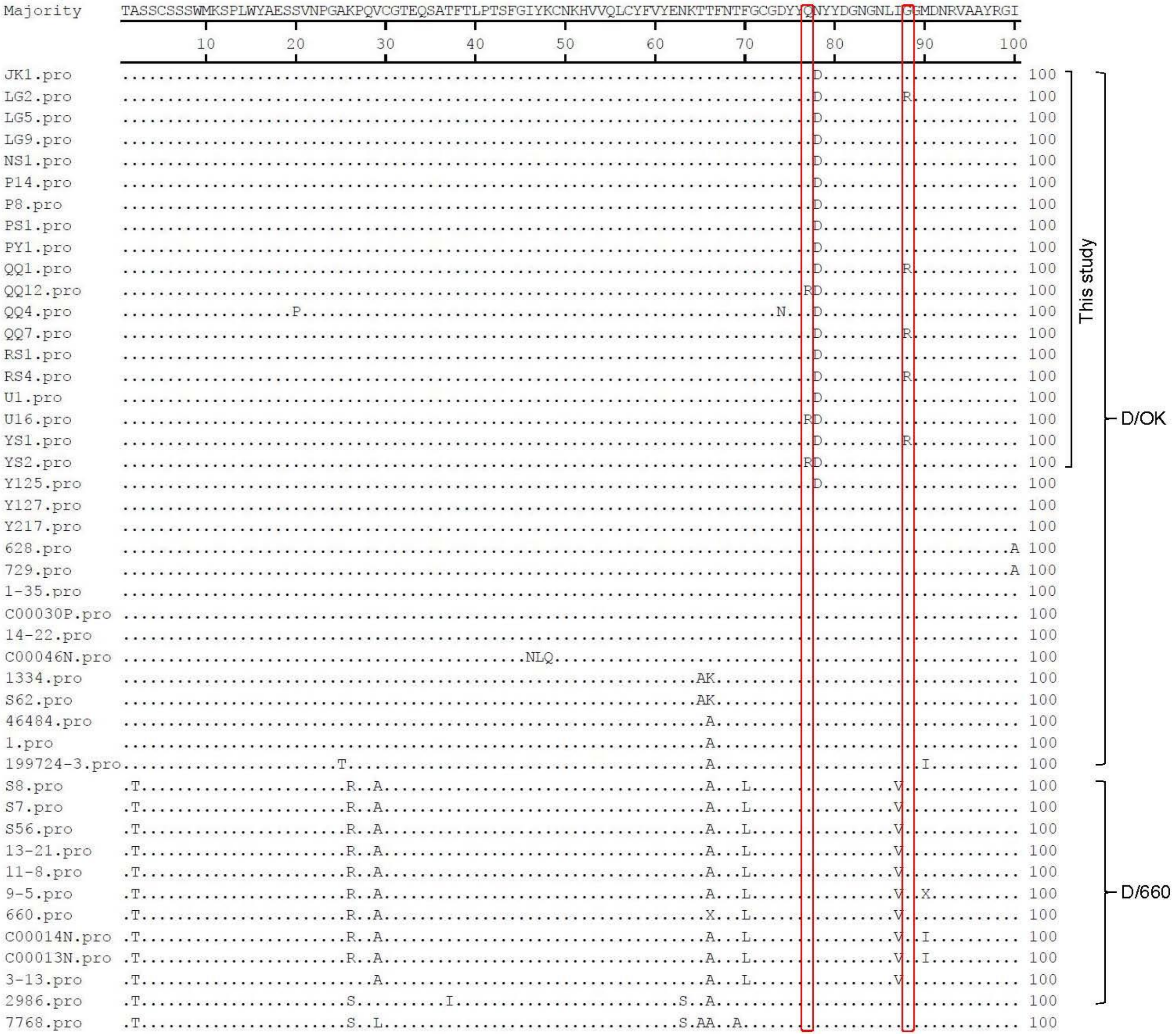
Majority
JK1.pro
LG2.pro
LG5.pro
LG9.pro
NS1.pro
P14.pro
P8.pro
PS1.pro
PY1.pro
QQ1.pro
QQ12.pro
QQ4.pro
QQ7.pro
RS1.pro
RS4.pro
U1.pro
U16.pro
YS1.pro
YS2.pro
Y125.pro
Y127.pro
Y217.pro
628.pro
729.pro
1-35.pro
C00030P.pro
14-22.pro
C00046N.pro
1334.pro
S62.pro
46484.pro
1.pro
199724-3.pro
S8.pro
S7.pro
S56.pro
13-21.pro
11-8.pro
9-5.pro
660.pro
C00014N.pro
C00013N.pro
3-13.pro
2986.pro
7768.pro
This study
D/OK
D/660

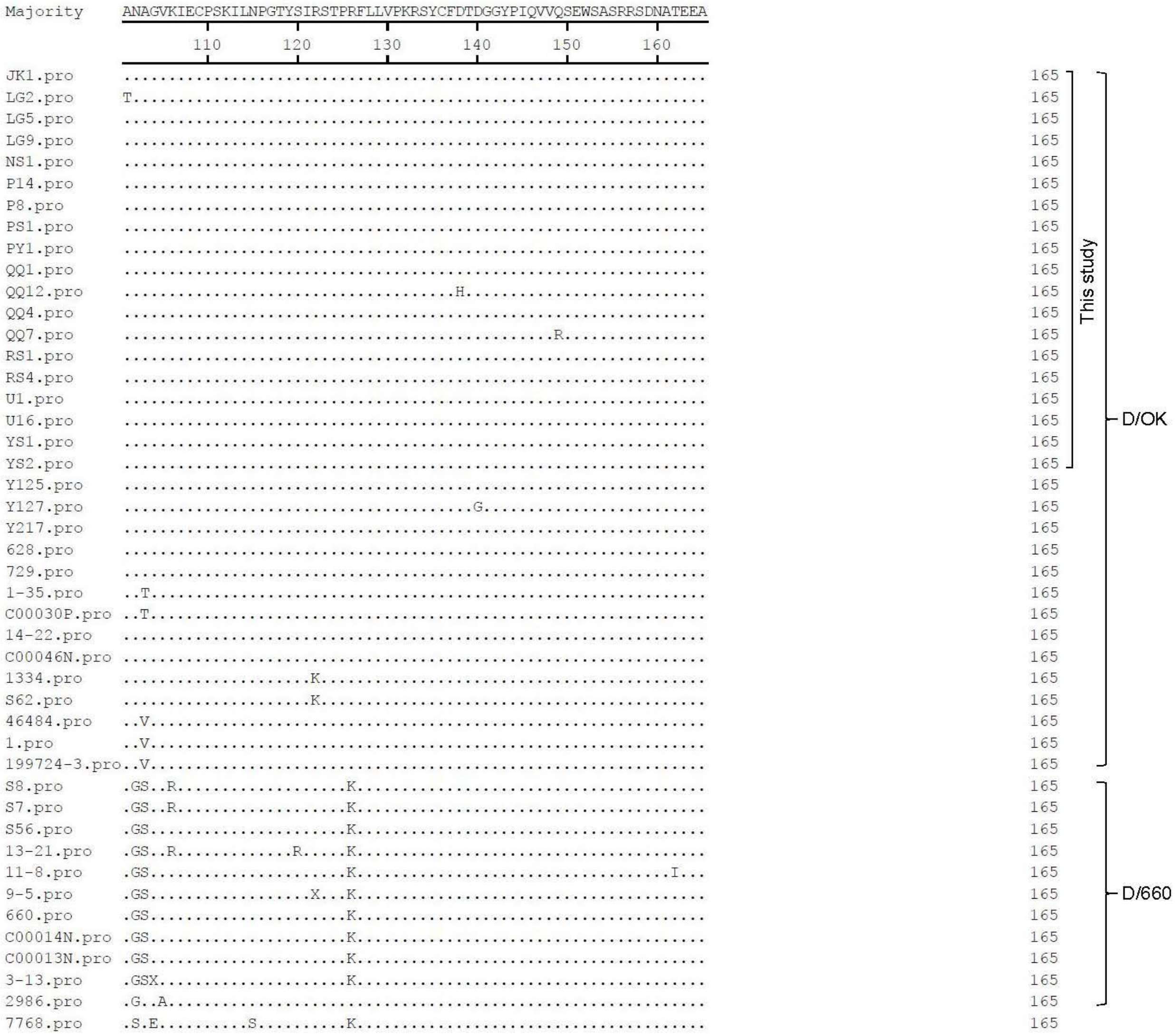

Technical Appendix Figure 2. Multiple sequence alignment results of the current IDV amino acid sequences (165 aa) and corresponding published reference sequences.
